# ^18^F-ASEM Imaging for Evaluating Atherosclerotic Plaques Linked to α7-Nicotinic Acetylcholine Receptor

**DOI:** 10.3389/fbioe.2021.684221

**Published:** 2021-07-01

**Authors:** Tao Yang, Dawei Wang, Xiangyi Chen, Yingkui Liang, Feng Guo, Chunxiao Wu, Liujun Jia, Zhihui Hou, Wenliang Li, ZuoXiang He, Xin Wang

**Affiliations:** ^1^Department of Cardiovascular Surgery, Fu Wai Hospital, Cardiovascular Institute, Chinese Academy of Medical Sciences and Peking Union Medical College, Beijing, China; ^2^Department of Nuclear Medicine, The Sixth Medical Center of PLA General Hospital, Beijing, China; ^3^Department of Nuclear Medicine, The First College of Clinical Medical Sciences, China Three Gorges University, Yichang, China; ^4^Yichang Central People’s Hospital, Yichang, China; ^5^School of Pharmacy, Jilin Medical University, Jilin City, China; ^6^Department of Nuclear Medicine, Beijing Tsinghua Changgung Hospital, Tsinghua University, Beijing, China

**Keywords:** ^18^F-ASEM, atherosclerotic plaques, α7-nicotinic acetylcholine receptor, PET/CT imaging, ^18^F-FDG

## Abstract

**Background:**

Atherosclerosis is a chronic vascular inflammatory procedure alongside with lipid efflux disorder and foam cell formation. α7-Nicotinic acetylcholine receptor (α7nAChR) is a gated-calcium transmembrane channel widely expressed in neuron and non-neuron cells, such as monocytes and macrophages, activated T cells, dendritic cells, and mast cells. ^18^F-ASEM is an inhibitor targeted to α7nAChR that had been successfully applied in nervous system diseases. Previous studies had highlighted that α7nAChR was related to the emergency of vulnerable atherosclerotic plaques with excess inflammation cells. Thus, ^18^F-ASEM could be a complementary diagnostic approach to atherosclerotic plaques.

**Materials and Methods:**

The synthesis of ASEM precursor and ^18^F-labeling had been performed successfully. We had established the ApoE^–/–^ mice atherosclerotic plaques model (fed with western diet) and New Zealand rabbits atherosclerotic models (balloon-sprained experiment and western diet). After damage of endothelial cells and primary plaque formation, ^18^F-ASEM imaging of atherosclerotic plaques linked to α7nAChR had been conducted. *In vivo* micro-PET/CT imaging of ApoE^–/–^ mice and the control group was performed 1 h after injection of ^18^F-ASEM (100–150 μCi); PET/CT imaging for rabbits with atherosclerotic plaques and control ones was also performed. Meanwhile, we also conducted CT scan on the abdominal aorta of these rabbits. After that, the animals were sacrificed, and the carotid and abdominal aorta were separately taken out for circular sections. The paraffin-embedded specimens were sectioned with 5 μm thickness and stained with hematoxylin–eosin (H&E) and oil red.

**Results:**

*In vivo* vessel binding of ^18^F-ASEM and α7nAChR expression in the model group with atherosclerosis plaques was significantly higher than that in the control group. PET/CT imaging successfully identified the atherosclerotic plaques in ApoE^–/–^ mice and model rabbits, whereas no obvious signals were detected in normal mice or rabbits. Compared with ^18^F-FDG, ^18^F-ASEM had more significant effect on the early monitoring of inflammation in carotid atherosclerotic plaques of ApoE^–/–^ mice and model rabbits. ^18^F-ASEM had relatively more palpable effect on the imaging of abdominal aorta with atherosclerosis in rabbits. H&E and oil red staining identified the formation of atherosclerotic plaques in model animals, which provided pathological basis for the evaluation of imaging effects.

**Conclusion:**

We first confirmed ^18^F-ASEM as radiotracer with good imaging properties for precise identification of atherosclerotic diseases.

## Introduction

Cardiovascular disease (CVD) is a serious threat to human health; its mortality is still the main cause of death (Writing Group Members et al., 2016). The integrity of vascular endothelial cells guaranteed the normal vascular function; thus, the disorder and dysfunction of endothelium are the precursors of more advanced lesions in arteries. Atherosclerosis (AS) is a main pathogenesis of CVD and an essential cause of major cardiovascular events (CVEs), accounting for more than 30% of total deaths worldwide (Anker et al., 2017). The progression of AS is a long-term pathological procedure characterized by the asymptomatic buildup of atherosclerotic plaques over decades that can result in sudden occurrence of fatal CVEs (Polonsky et al., 2017). Therefore, it is of great importance to detect atherosclerotic plaques at the early stage and to perform therapeutic approach to prevent the occurrence of CVEs. The methods to identify high-risk patients range from clinical assessment of risk factors to noninvasive imaging of atherosclerotic plaques like computed tomography (CT), intravascular ultrasound (IVUS), optical coherence tomography (OCT), and magnetic resonance imaging (MRI) (Piepoli et al., 2016; Boswijk et al., 2017). Although these imaging modalities can provide evidence of lumen stenosis and display atherosclerotic plaques features, their resolution is still insufficient, therefore could not identify atherosclerotic plaques in the early stage. Positron emission computed tomography (PET) as the most advanced molecular imaging protocol, however, can make up for the shortcomings mentioned above: It cannot only visualize the pathogenesis of atherosclerotic plaques in the early stage of lesions with high sensitivity but also directly show the progression of lesions in order to guide the early intervention of AS (Evans et al., 2016; Creager et al., 2019).

The homomeric α7 subtype of nicotinic acetylcholine receptors (α7nAChR) is a class of ligand-gated ion channels distributed throughout the brain, and the crucial role of α7nAChR in a number of specific systems and diseases has been extensively reviewed (Hurst et al., 2013; Bertrand and Terry, 2018). Its abnormalities has been implicated in a wide variety of central nervous system disorders (Kalkman and Feuerbach, 2016). Only recently, however, several studies have found α7nAChR to be a major contributor in the formation of atherosclerosis and is becoming the target for early identification of atherosclerotic plaques (Chen et al., 2016; Wang et al., 2017). Although α7nAChR appears to be involved in the pathophysiology of AS, until now, its exact role in this disease remains unknown. It might be concluded from the information above that manipulation of α7nAChR would have an effect on the formation of atherosclerotic plaques. To slow down pathophysiological progressing and troubled recruitment of immune cells and immune cells cytokines along with the reconstruction of arteries, the α7nAChR integrated inflammation into atherosclerosis occurrence, especially in unstable plaques, with characteristics of thin fibrotic cap and awakened immune cells. Thus, reliable identification of α7nAChR by *in vivo* imaging is of great importance to investigate the exact role of α7nAChR in AS before any theranostic approach in human setting can be justified. The exploration of α7nAChR by PET is a highly promising way not only to better understand the changes associated with the progression of AS but also to improve the development of new therapeutic strategies. Therefore, a highly specific radiotracer could be used to image differences in receptor density in different disease stages and expand our knowledge of α7nAChR’s contribution to the pathophysiology of AS.

**FIGURE 1:**
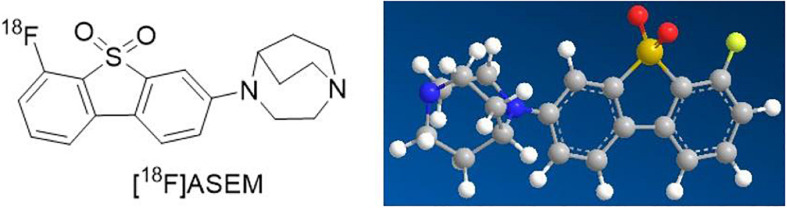
The chemical structure and three-dimensional structure of ^18^F-ASEM.

For these reasons mentioned above, α7nAChR, as a significant therapeutic target, has received increasing interest in the fields of science and clinical medicine. Therefore, great progress in quantity and quality of α7nAChR radioligands has been made over the past decades (Chalon et al., 2015). Imaging and quantifying of α7nAChR with PET might offer a noninvasive way to critically facilitate the early diagnosis of AS and provide an insight into its role in atherosclerotic plaques. Up to the present, it has been reported that the best ^18^F-labeled probe in α7nAChR is dibenzo[b,d] thiophendioxide derivative, named ASEM (Teodoro et al., 2018; Figure 1). ^18^F-ASEM was developed by Gao et al., with high specific affinity for α7nAChR (*K*_*i*_ = 0.4 nM), has been carried out in human studies, and are proved to be the only α7nAChR PET radioligand available to humans with promising imaging characteristic (Gao et al., 2013; Hillmer et al., 2017; Coughlin et al., 2018). However, ^18^F-ASEM has thus far been studied only for its use in neuroimaging but not in the imaging of vessels. The ideal features for a certain tracer for imaging of central nervous system receptors differ from those required for the imaging of receptors expression in the vasculature (Boswijk et al., 2017). Therefore, based on animal models (ApoE^–/–^ mice and atherosclerotic New Zealand rabbits), the present study reports the first PET/CT imaging with ^18^F-ASEM, making it feasible to image atherosclerotic plaques and to evaluate the vulnerability of plaques toward rupture. We also made pathological examination of AS to verify the reliability of ^18^F-ASEM in the early identification of atherosclerotic plaques.

## Materials and Methods

ApoE^–/–^ mice were purchased from Qingzilan Technology Co., Ltd. (Nanjing, China), and New Zealand rabbits were acquired from Fuwai Hospital (Beijing, China). C-^18^ light Sep-Pak cartridges and QMA light ion-exchange cartridges were obtained from Waters (Milford, MA, United States). The ^18^F isotope was acquired through Jiangsu Huayi Technology Co., Ltd. (Changshu, China).

The scaffolds and ^18^F-labeled ASEM were synthesized and followed the descriptions in several previous studies (Gao et al., 2013; Wang et al., 2021). Briefly, the ortho-substitution amino was countered with nucleophilic radio-fluorination in the last Sandmeyer reaction. Then, the product was purified by high-performance liquid chromatography (HPLC) and diluted through a C^18^ column. The radiochemical purity was > 99%.

### Animal Models

Animal care and experimental procedures were approved by Animal Care Committee of Fuwai Hospital.

#### Establishment of Carotid Atherosclerotic Plaques Model

ApoE^–/–^ mice (*n* = 4; weight, 25–30 g) and normal mice (*n* = 4; weight, 25–30 g) were randomly selected; the chosen ApoE^–/–^ mice were fed with 21% fat and 0.15% cholesterol supplement for 11–12 weeks.

#### Establishment of Abdominal Atherosclerotic Plaques Model

**FIGURE 2:**
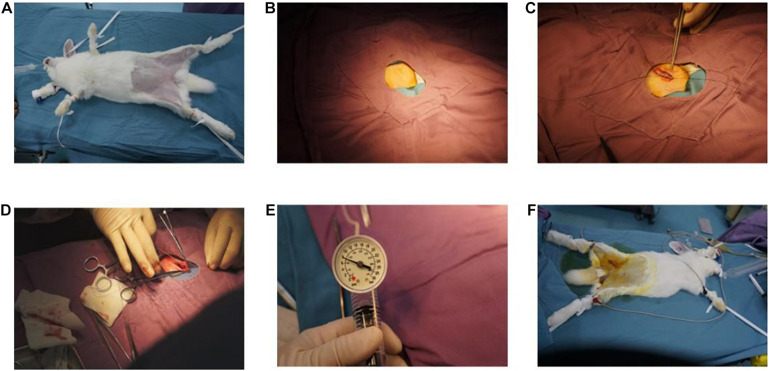
Balloon sprained and injured experiment: **(A)** immobilization of animals, anesthesia, and skin preparation. **(B)** Disinfect, spread towel, and expose incision. **(C)** Free femoral artery. **(D)** Balloon catheter was implanted. **(E)** The balloon was filled with gas and was pulled repeatedly in abdominal aorta for three to five times. **(F)** The incision was sutured, and the operation was finished.

New Zealand rabbits (*n* = 4; weight, 2.5–3.0 kg) were fed with high-fat diet for 14 weeks. The balloon-sprained and injured experiment was carried out at the third week after 2 weeks of high-fat diet. First, rabbits were anesthetized by 10% chloral hydrate, a 3-cm incision was conducted in the right inguinal area to expose the right femoral artery; then, the right femoral artery was punctured by Seldinger technique. Second, an angiographic catheter with double lumen balloon was sent to the aortic arch under fluoroscopy. Third, the balloon was bulged and pulled down to the bifurcation of the distal iliac artery of the abdominal aorta for three times. Lastly, the catheter and sheath were removed, and the operational incisions were sutured (Figure 2).

#### *In vivo* PET/CT Imaging

##### Mice PET Imaging

The high-performance liquid chromatography (HPLC)-purified ^18^F-ASEM [20 μCi, dissolved in 0.2 ml normal saline, containing 5% dimethyl sulfoxide (DMSO)] was injected into tail vein of ApoE^–/–^ mice and normal mice. ApoE^–/–^ mice, positioned prone position, were anesthetized (1–2% isoflurane) through inhalation. After anesthesia, mice were injected with 5.56 MBq (150 μCi) ^18^F-ASEM through the tail vein and underwent a dynamic imaging for 1 h. Dynamic PET scanning were carried out at 5, 10, 15, 30, and 60 min after injection, and biological distributions were analyzed as previously described by our group (Wang et al., 2021). Briefly, the regions of interest (ROIs) were cycled from 3D ordered subset expectation maximization (OSEM 3D) reconstruction images, then % ID/g value was calculated by Inveon Research Workplace 4.0 (IRW4.0).

##### New Zealand Rabbits PET Imaging

First, rabbits (*n* = 4) were anesthetized by 10% chloral hydrate (2 ml/kg). Then, ^18^F-ASEM solution (74 MBq in 0.2 ml normal saline) was injected into the right ear vein of rabbits. After that, the imaging procedure was conducted 30 min after injection of ^18^F-ASEM and lasted for 10 min performed by PET/CT (Discovery IQ 2.0 GE). The injection of ^18^F-FDG (74 MBq in 0.2 ml normal saline) and PET/CT imaging were performed in the same strategy on the next day. The reconstruction mode was super iterative. The % ID/g value was worked out in the same protocol as mentioned above through IRW 4.0 software.

#### *In vivo* CT Scanning

All rabbits were treated with a second-generation dual-source CT scan (SOMATOM Definition Flash; Siemens Healthcare, Forchheim, Germany). The scan area ranged from the aortic arch to the level of bifurcation of the iliac artery. X-ray tube current was adjusted individually. For contrast medium enhancement, automated bolus tracking was used in a region of interest within the ascending aorta, with a signal attenuation trigger threshold of 100 HU, 2-s postponed. The main scanning parameters were as follows: 0.6 mm individual detector width, 280 ms gantry rotation time, 0.20–0.50 pitch, and 200–250 mm field of view for raw image reconstruction. We used a triple-phase contrast medium injection protocol, which consisted of 5–6 ml of undiluted contrast agent (Iopromide, 370 mgI/ml; Bayer Healthcare, Berlin, Germany) followed by a 3-ml 30%:70% mixture of contrast medium and saline and a 3-ml saline chaser bolus, all injected with flowrates of 1–2 ml/s.

All original imaging date were retrospectively analyzed on the workstation (Deep Blue, ADW4.6, GE Healthcare, Milwaukee, WI, United States) by two experienced radiologists using axial images, multiplanar reconstructions, and maximum intensity projections, as appropriate. The presence of atherosclerosis in the aorta within the scan area was analyzed. Any disagreements between the two readers were solved by consensus.

### Tissue Preparation and Histological Measurement

After anesthetized, the mice and rabbits were sacrificed for histological measurements and experiments. Carotid arteries of model mice and abdominal aortas of model rabbits were perfused with heparin saline, harvested under aseptic conditions, and fixed in 4% polyformaldehyde. The full length of the carotid arteries and abdominal aortas was used for H&E staining and *en face* analysis by red “O” staining.

#### Hematoxylin–Eosin Staining

First, paraffin-embedded slices were dewaxed in water. The slices were successively put into xylene (10 min), anhydrous ethanol (10 min), anhydrous ethanol (5 min), 95% (5 min) alcohol, 90% alcohol (5 min), 80% alcohol (5 min), 70% alcohol (5 min), and distilled water (5 min) to wash step by step. Then, slices were embedded with hematoxylin dye for 8 min, then washed with flowing water. The slices were differentiated with 1% hydrochloric acid alcohol for 10 s, washed with flowing water, retreated with 0.6% ammonium hydroxide until turning blue, then washed with running water. After hematoxylin staining the nucleus, the slices were stained for 1–3 min in eosin dye solution for eosin staining the cytoplasm. The slices were dehydrated and were transparent in 95% alcohol I, 5 min–95% alcohol, 5 min–anhydrous ethanol, 5 min–anhydrous ethanol, and 5 min–xylene. The slices were slightly dried from xylene and sealed with neutral gum for image acquisition and analysis.

#### Red “O” Staining

First, carotid arteries and abdominal aortas were prepared for frozen sectioning. Then, several frozen sections of arteries were rewarmed at room temperature, treated with fixative (G1101, Servicebio, Wuhan, China) for 15 min, then washed with running water and dried. Then, the slices were immersed into the oil red dye solution (G1016, Servicebio, Wuhan, China), kept for 10 min in a dark place, washed with distilled water, slightly differentiated with 75% alcohol, and washed with distilled water again. Afterward, the slices were dyed with hematoxylin solution (G1004, Servicebio, Wuhan, China) for 5 min, washed with flowing water, differentiated with differentiation solution then washed with flowing water again, retreated with returned blue solution (G1005-4, Servicebio, Wuhan, China), and washed with running water. Finally, they were sealed with glycerin gelatin (G1402, Servicebio, Wuhan, China) and examined with a microscope.

### Statistical Analysis

SPSS Statistics v24 (IBM Corp., Armonk, NY, United States) software was used. All data were expressed as mean ± SD. *P* < 0.05 were considered statistically significant.

## Results

### PET/CT Imaging

**FIGURE 3:**
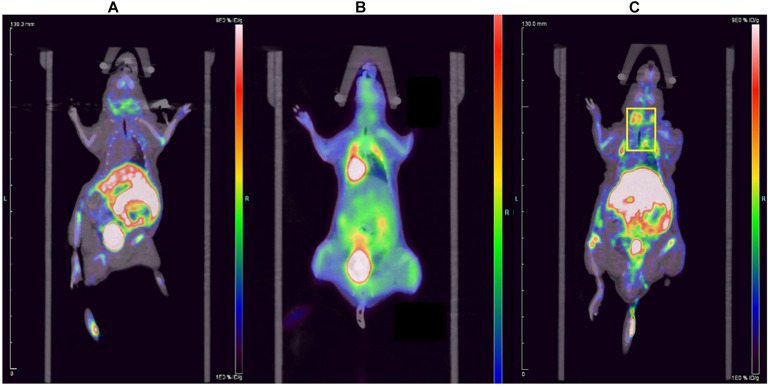
Comparison of the **(A)** normal mouse, **(B)** ^18^F-FDG imaging of carotid plaques in ApoE^–/–^ mouse and **(C)** ^18^F-ASEM imaging of carotid plaques in ApoE^–/–^ mouse.

The ^18^F-ASEM micro-PET/CT images of ApoE^–/–^ mouse and normal mouse are shown in Figure 3. Compared to ^18^F-FDG, the imaging agent was more remarkable in the carotid artery of ApoE^–/–^ mouse (Figure 3C; yellow frame in the right-side figure below), which indicates that ^18^F-ASEM is superior to ^18^F-FDG in the early detection of atherosclerotic plaques in carotid plaques of ApoE^–/–^ mice. ^18^F-ASEM can specifically bind to α7nAChR in the model group, whereas it had no signal in the normal group.

**FIGURE 4:**
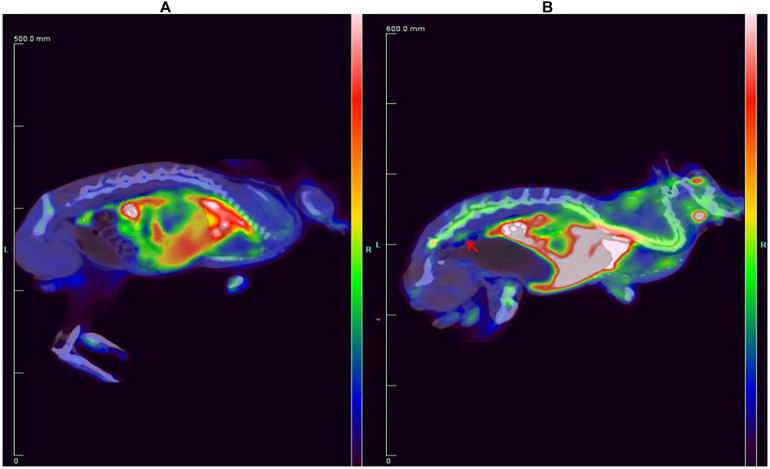
Comparison of the New Zealand rabbit **(A)** treated with ^18^F-FDG and **(B)** treated with ^18^F-ASEM, in which the red arrow shows that ^18^F-ASEM can recognize atherosclerotic plaques at the lower segment of abdominal aorta in the early stage.

The ^18^F-ASEM and ^18^F-FDG PET/CT images of the rabbit are shown in Figure 4. Compared to the rabbit treated with ^18^F-FDG (Figure 4), ^18^F-ASEM showed a remarkable uptake in plaques in the site of abdominal arterial scratches zones (red arrow). This indicates that the abdominal atherosclerotic plaques can be obviously recognized with ^18^F-ASEM, which is better than ^18^F-FDG in the early stage of atherosclerotic lesions.

### CT Imaging of New Zealand Rabbits

**FIGURE 5:**
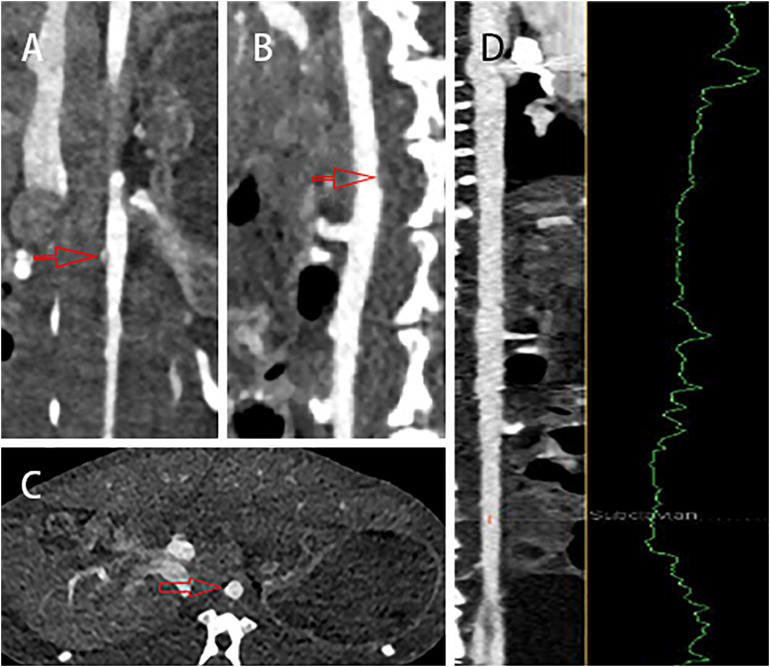
The **(A)** coronal, **(B)** sagittal, and **(C)** cross-sectional CT scans showed that the wall of abdominal aorta was thickened and irregular (red arrow), but there was no obvious sign of atherosclerotic plaques. **(D)** In addition, there was no significant stenosis in the whole abdominal aorta.

The coronal, sagittal, and cross-sectional CT scan imaging showed that the wall of abdominal aorta was thickened and irregular (red arrow). However, there was no significant stenosis or notable sign related with atherosclerotic plaques (Figure 5). Moreover, the stenosis rate analysis software we used quantitatively analyzed the diameter of the abdominal aorta and showed that there was no significant stenosis in the whole abdominal aorta. All information mentioned above completely demonstrated that CT imaging could not recognize atherosclerotic plaques in the early stage.

### Biological Distribution Studies of Mice

**FIGURE 6:**
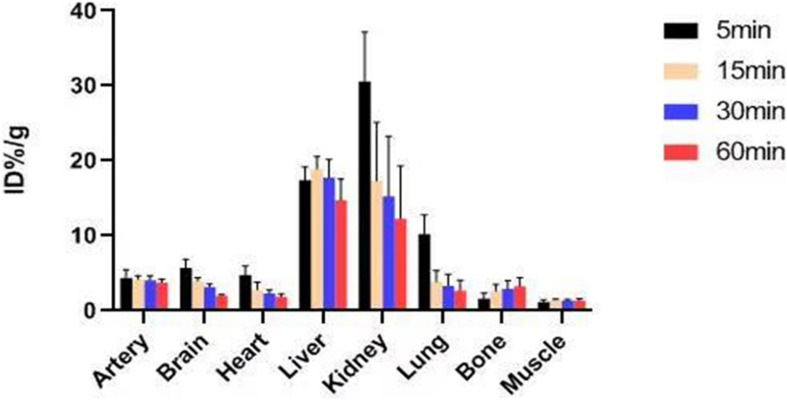
Biological distribution map and table of normal mice.

**FIGURE 7:**
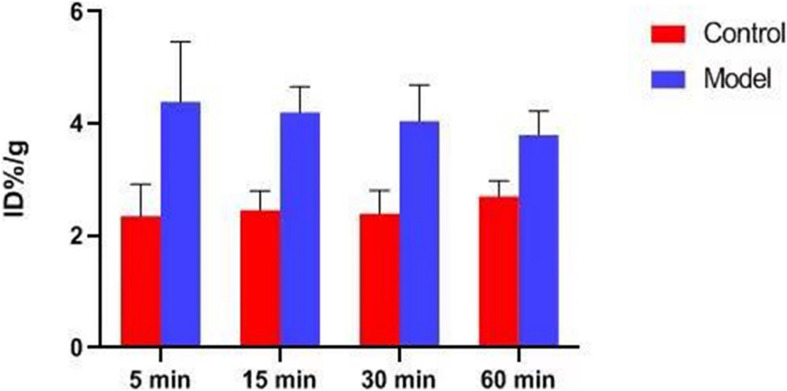
Biological distribution rate of mice between ApoE^–/–^ mice and mice in the control group.

In previous studies, the *K*_*i*_-value of ASEM was measured (*K*_*i*_ = 0.4 nM), and blocking experiment also proved that ASEM is specifically bound to α7nAChR (Gao et al., 2013; Wang et al., 2018). The biological distribution of the tracer in normal mice is shown in Figure 6. The result demonstrated that the highest accumulation of radiotracer was observed in the liver and kidney, with a peak uptake at 15 min in the liver and 5 min in the kidney after injection. The lowest uptake in the muscle and the excellent signal-to-noise ratio contributed to an obvious insight of plaque lesions. The uptake of ^18^F-ASEM in ApoE^–/–^ mice was much higher than in mice of the control group, which was also slightly decreased during this examination in the model group (Figures 6, 7).

### Hematoxylin–Eosin Staining and Red “O” Staining

**FIGURE 8:**
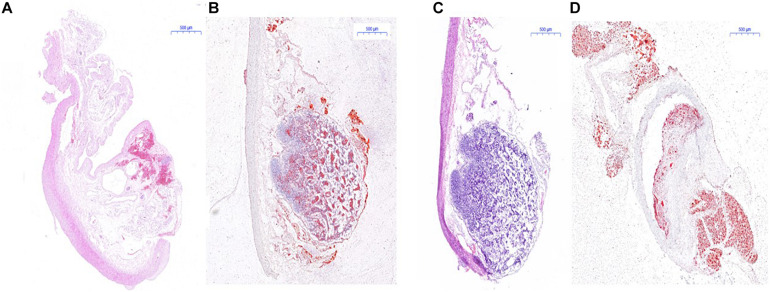
Histological measurement of both ApoE^–/–^ mice and rabbit. **(A)** H&E staining for the determination of atherosclerotic plaques area of rabbit; **(B)** red “O” staining for the determination of atherosclerotic plaques area of rabbit; **(C)** H&E staining of atherosclerotic plaques area of mice; **(D)** red “O” staining of atherosclerotic plaques area of mice.

**FIGURE 9:**
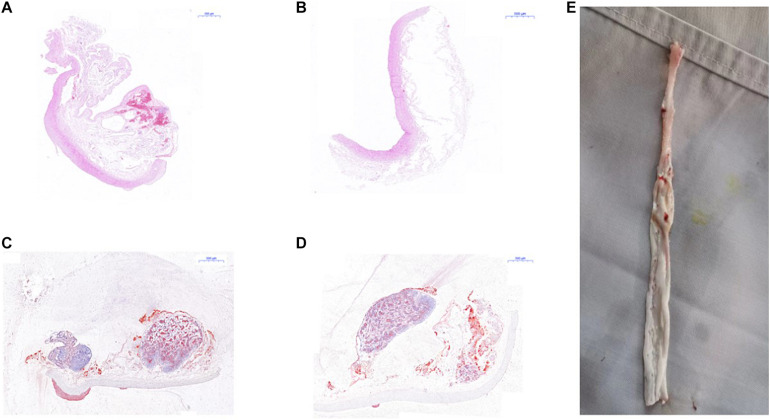
Pathological examination of abdominal aorta in rabbits. **(A)** H&E staining indicated atherosclerotic plaques formation in the abdominal aorta of New Zealand rabbits in the model group, **(B)** while there was no plaques formation in abdominal aorta of rabbits in the control group; **(C)** red “O” staining suggested atherosclerotic plaques formation in the abdominal aorta of New Zealand rabbits in the model group, **(D)** while there was no sign of plaques formation in the abdominal aorta of rabbits in the control group. **(E)** The specimen of the abdominal aorta of rabbits in the model group showed that the wall of the artery was more thickened, less smooth, and had remarkable atherosclerotic plaques formation.

The result of H&E staining and red “O” staining of the carotid artery of mice and the abdominal aorta of rabbit is shown in Figures 8, 9. In H&E staining of plaques in both mouse and rabbit (Figures 8A,C), the normal vascular structure had been destroyed instead of atherosclerotic plaque lesion, which fully confirmed the successful establishment of an atherosclerotic plaques model in ApoE^–/–^ mice and New Zealand rabbits. Red “O” staining of arteries (Figures 8B,D) also showed that the area of atherosclerotic plaques lesion was bright red as a result of lipid accumulation and foam cell formation, whereas it was blue in the nucleus and colorless in the stroma. The section further demonstrated the formation of typical atherosclerotic plaques in carotid arteries in ApoE^–/–^ mice, which reflects the presence of lipid content in atherosclerotic plaques. These stable models finally confirmed the imaging effect of PET/CT and further explains the early formation of atherosclerotic plaques.

## Discussion

The nervous and immune systems are the crucial footstone of human beings after innumerable iterations of evolution, which play an important role in intricate regulation and adjustment of homeostasis. The reflex of benefit tending and harm avoiding are the most basic reaction of vertebrates. The cholinergic system is an integral part of the human beings, which also plays a significant role in metabolic activities such as proliferation, apoptosis, assimilation, and dissimilation. Acetylcholine is an important neurotransmitter excrete from the cytoplasm to synaptic space through synaptic vesicles (Luo et al., 2020). Nicotinic acetylcholine receptors and muscarinic acetylcholine receptors can be stimulated by free acetylcholine. Nicotinic acetylcholine receptors are pentameric ligand-gated ion transmembrane protein with homologous or heterogeneous nAChR subtype participating in physiological responses to acetylcholine in neuronal and non-neuronal cells. The α7nAChR with five homologous α7 subunits that combine five special-binding sites was first found in the mammalian cortex. The α7nAChRs are also expressed in non-neuronal cells such as B cells, T cells, mononuclear macrophages, vascular endothelium, chondrocytes, and vascular smooth muscle cells (Courties et al., 2020). Activating state of α7nAChR may have a reverse signal transduction function, which results in anti- and proinflammation abilities (Hoover, 2017; Vieira-Alves et al., 2020).

Atherosclerotic plaques formation is a complex process that involves inflammation, endothelial cell dysfunction, apoptosis, and advanced microcalcification (Tarkin et al., 2014). CT scanning could show the abnormal morphology directly (Figures 5A–C), assessing the severity of the arterial stenosis. However, the stenosis rate analysis software that we used failed to alert thickened and irregular abdominal aorta of the rabbits (Figure 5D). Although ^18^F-FDG and ^18^F-NaF had significant diagnostic usages, for example, ^18^F-FDG PET imaging could detect the nonspecificity inflammatory responses, and ^18^F-NaF PET imaging has the capability of detecting advanced microcalcification, there still remains a challenge to timely predict the risk of rupture for a specific atherosclerotic plaque, a thrombotic trigger tightly linked to inflammation. In our study, we used ^18^F-FDG and ^18^F-ASEM as a possible plaque tracer. There is slight ^18^F-FDG uptake in the carotid artery of ApoE^–/–^ mouse, whereas there is more ^18^F-ASEM gathering in the same animal model. Although accumulation of ASEM in the arteries was slightly decreased during this examination (Figures 6, 7), the uptake of ^18^F-ASEM in the arteries of ApoE^–/–^ mice were still much higher than that in the mice in the control group at 5 min (4.39 ± 1.08 ID%/g vs. 2.35 ± 0.58 ID%/g), 15 min (4.20 ± 0.46 ID%/g vs. 2.46 ± 0.34 ID%/g), 30 min (4.05 ± 0.64 ID%/g vs. 2.40 ± 0.41 ID%/g), and 60 min (3.80 ± 0.43 ID%/g vs. 2.70 ± 0.28 ID%/g) after injection (Figure 7). The plaques had been verified by H&E staining and red “O” staining (Figures 8C,D). Fortunately, we had found the same phenomenon in the abdominal aorta of rabbits in the model group (Figures 4, Figures 8A,B). Pathological examination of the abdominal aorta in normal rabbits also demonstrated that the direct injury has promoted the formation of aortic atherosclerotic plaques (Figure 9). Previous studies had demonstrated that α7nAChR in vasculature is closely associated with atherosclerotic plaques (Boswijk et al., 2017). The α7nAChR is upregulated in several non-neuronal cells involved in the prime and progression of plaques, including mononuclear macrophage, lymphocytes, and vascular endothelial and smooth muscle cells (Vieira-Alves et al., 2020). It is also possible that α7nAChR plays different roles in different stages of atherosclerosis and other chronic inflammatory diseases. Thus, imaging of α7nAChR *in vivo* is a crucial step within the context of the potential therapeutic use of these receptors in the future, and it would provide an effective tool to identify atherosclerotic plaques that are prone to rupture. A number of noninvasive imaging techniques have been developed to evaluate the vascular wall in an attempt to identify atherosclerotic plaques that are prone to rupture, which are the so-called vulnerable plaques. Because morphological imaging has some limitations in early diagnosis of these plaques, molecular imaging has emerged as a novel tool to identify events that happened at molecular level critical for the pathogenesis of atherosclerotic plaques. Among them, PET radiotracers would be more ideal for identifying those plaques (Zhao et al., 2020). Therefore, it becomes extremely significant to find appropriate PET molecular probes that can specifically bind to α7nAChR in vascular plaques.

In the search for a novel PET tracer that can help identify patients with relatively higher risk of developing complications of atherosclerosis, α7nAChR PET tracers would play a significant role. Until now, the best ^18^F-labeled molecular probe for α7nAChR reported in the literature is the dibenzo[b,d] thiophendioxide derivative, named ASEM, which shows the greatest potential for translation to imaging atherosclerosis. ASEM was synthesized by Gao et al., with high specific affinity for α7nAChR (*K*_*i*_ = 0.4 nM) and good specificity (*BP*_*ND*_ = 3.9–6.6) (Gao et al., 2013; Wang et al., 2018). The biological distribution shows excellent advantage, as is shown in our results. ^18^F-ASEM has been carried out in human studies and are proven to be the only α7nAChR PET radioligand available to humans with promising pharmacokinetic and imaging characteristics (Wong et al., 2014; Aharonson et al., 2020). However, ^18^F-ASEM has thus far been studied for only its use in neuroimaging but not in the imaging of vasculature, mainly because the ideal conditions required for radioligand imaging of α7nAChR in the nervous system are different from those of the receptors in blood vessels. Therefore, the evaluation of the vulnerability of atherosclerotic plaques by PET/CT imaging, with an appropriate probe such as ^18^F-ASEM for α7nAChR in blood vessels, has potential applications in clinical setting.

In the present study, we established atherosclerotic plaques models of carotid arteries in ApoE^–/–^ mice and abdominal aorta in New Zealand rabbits and reported the first PET/CT imaging conducted with ^18^F-ASEM, which makes it feasible to image atherosclerotic plaques and to evaluate the vulnerability of plaques toward rupture. Although CT imaging modality can provide evidence of lumen stenosis and display features of remarkable plaques, its resolution is still insufficient, therefore could not identify atherosclerotic plaques in the earlier stage. ^18^F-ASEM had more flexibility in inflammatory disease monitoring, which not only benefit from the specificity to α7nAChR in blood vessels but also originate from better pharmacokinetics compared to nonspecificity ^18^F-FDG (Zhang et al., 2019; Liu et al., 2020). Our results indicated that not only CT imaging could not recognize atherosclerotic plaques in the early stage but also PET/CT imaging with ^18^F-ASEM is more sensitive to detect inflammation and might present stronger signal intensity of inflamed atherosclerotic plaques compared to the classic ^18^F-FDG-based nuclear medicine imaging modality. In addition, we also made pathological examination of atherosclerosis to verify the reliability of ^18^F-ASEM in the identification of atherosclerotic plaques.

From the present study, we know that ^18^F-ASEM might be also used to evaluate the effect of α7nAChR targeting strategies in the early diagnosis of atherosclerosis. Further clinical studies will answer the question as to whether ^18^F-ASEM, as the best molecular probe for α7nAChR reported in the literature, can be reliably applied clinically in noninvasive, early diagnosis and monitoring of the vulnerable atherosclerotic plaques of patients who would suffer from acute coronary syndrome. Achieving the early warning of CVEs, thus, greatly reduce the associated morbidity and mortality of patients with CADs all over the world.

## Conclusion

The present study supported the feasibility of ^18^F-ASEM as the α7nAChR targeting tracer to detect the inflammation in the formation of atherosclerosis, which holds the potential to assess the vulnerability of plaques. Therefore, ^18^F-ASEM, as a molecular probe, has the potential to be an appropriate α7nAChR-targeted imaging radiotracer for the early identification of atherosclerotic plaques and other chronic inflammation diseases. Further studies on this radioligand series are still ongoing.

## Data Availability Statement

The raw data supporting the conclusions of this article will be made available by the authors, without undue reservation.

## Ethics Statement

The animal study was reviewed and approved by the Animal Care Committee of Fu Wai Hospital.

## Author Contributions

XW, ZHe, and WL designed the experiments. TY, DW, YL, FG, CW, LJ, and ZHo performed the experiments. TY, DW, and XC analyzed the data and wrote the manuscript. All authors contributed to the article and approved the submitted version.

## Conflict of Interest

The authors declare that the research was conducted in the absence of any commercial or financial relationships that could be constructed as a potential conflict of interest.

## References

[B1] Aharonson V. Seedat N. Israeli-Korn S. Hassin-Baer S. Postema M. Yahalom G. (2020). Automated stage discrimination of Parkinson’s disease. *BIO Integr.* 1 55–63. 10.15212/bioi-2020-0006

[B2] Anker S. Asselbergs F. W. Brobert G. Vardas P. Grobbee D. E. Cronin M. (2017). Big data in cardiovascular disease. *Eur. Heart J.* 38 1863–1865. 10.1093/eurheartj/ehx283 28863460

[B3] Bertrand D. Terry A. Jr. (2018). The wonderland of neuronal nicotinic acetylcholine receptors. *Biochem. Pharmacol.* 151 214–225. 10.1016/j.bcp.2017.12.008 29248596

[B4] Boswijk E. Bauwens M. Mottaghy F. M. Wildberger J. E. Bucerius J. (2017). Potential of α7 nicotinic acetylcholine receptor PET imaging in atherosclerosis. *Methods* 130 90–104. 10.1016/j.ymeth.2017.06.008 28602809

[B5] Chalon S. Vercouillie J. Guilloteau D. Suzenet F. Routier S. (2015). PET tracers for imaging brain α7 nicotinic receptors: an update. *Chem. Commun. (Camb.)* 51 14826–14831. 10.1039/c5cc04536c 26359819

[B6] Chen L. Liu D.-H. Zhang X. Zhang E.-H. Liu C. Su D.-F. (2016). Baroreflex deficiency aggravates atherosclerosis via α7 nicotinic acetylcholine receptor in mice. *Vascul. Pharmacol.* 87 92–99. 10.1016/j.vph.2016.08.008 27568460

[B7] Coughlin J. M. Du Y. Rosenthal H. B. Slania S. Koo S. M. Park A. (2018). The distribution of the alpha7 nicotinic acetylcholine receptor in healthy aging: an in vivo positron emission tomography study with [18F] ASEM. *Neuroimage* 165 118–124. 10.1016/j.neuroimage.2017.10.009 28993233 PMC5738927

[B8] Courties A. Do A. Leite S. Legris M. Sudre L. Pigenet A. (2020). The role of the non-neuronal cholinergic system in inflammation and degradation processes in osteoarthritis. *Arthritis Rheumatol.* 72 2072–2082. 10.1002/art.41429 32638534

[B9] Creager M. D. Hohl T. Hutcheson J. D. Moss A. J. Schlotter F. Blaser M. C. (2019). ^18^F-fluoride signal amplification identifies microcalcifications associated with atherosclerotic plaque instability in positron emission tomography/computed tomography images. *Circ. Cardiovasc. Imaging* 12:e007835. 10.1161/CIRCIMAGING.118.007835 30642216 PMC6338081

[B10] Evans N. R. Tarkin J. M. Chowdhury M. M. Warburton E. A. Rudd J. H. (2016). PET imaging of atherosclerotic disease: advancing plaque assessment from anatomy to pathophysiology. *Curr. Atheroscler. Rep.* 18:30. 10.1007/s11883-016-0584-3 27108163 PMC4842219

[B11] Gao Y. Kellar K. J. Yasuda R. P. Tran T. Xiao Y. Dannals R. F. (2013). Derivatives of dibenzothiophene for positron emission tomography imaging of α7-nicotinic acetylcholine receptors. *J. Med. Chem.* 56 7574–7589. 10.1021/jm401184f 24050653 PMC3866913

[B12] Hillmer A. T. Li S. Zheng M.-Q. Scheunemann M. Lin S.-F. Nabulsi N. (2017). PET imaging of α 7 nicotinic acetylcholine receptors: a comparative study of [18 F] ASEM and [18 F] DBT-10 in nonhuman primates, and further evaluation of [18 F] ASEM in humans. *Eur. J. Nucl. Med. Mol. Imaging* 44 1042–1050. 10.1007/s00259-017-3621-8 28120003 PMC5400702

[B13] Hoover D. B. (2017). Cholinergic modulation of the immune system presents new approaches for treating inflammation. *Pharmacol. Ther.* 179 1–16. 10.1016/j.pharmthera.2017.05.002 28529069 PMC5651192

[B14] Hurst R. Rollema H. Bertrand D. (2013). Nicotinic acetylcholine receptors: from basic science to therapeutics. *Pharmacol. Ther.* 137 22–54. 10.1016/j.pharmthera.2012.08.012 22925690

[B15] Kalkman H. O. Feuerbach D. (2016). Modulatory effects of α7 nAChRs on the immune system and its relevance for CNS disorders. *Cell. Mol. Life Sci.* 73 2511–2530. 10.1007/s00018-016-2175-4 26979166 PMC4894934

[B16] Liu C. Jiao D. Liu Z. (2020). Artificial intelligence (AI)-aided disease prediction. *BIO Integr.* 1 130–136. 10.15212/bioi-2020-0017

[B17] Luo X. Lauwers M. Layer P. G. Wen C. (2020). Non-neuronal role of acetylcholinesterase in bone development and degeneration. *Front. Cell Dev. Biol.* 8:620543. 10.3389/fcell.2020.620543 33585459 PMC7876280

[B18] Piepoli M. F. Hoes A. W. Agewall S. Albus C. Brotons C. Catapano A. L. (2016). 2016 European guidelines on cardiovascular disease prevention in clinical practice: the sixth joint task force of the European society of cardiology and other societies on cardiovascular disease prevention in clinical practice (constituted by representatives of 10 societies and by invited experts) developed with the special contribution of the European association for cardiovascular prevention & rehabilitation (EACPR). *Eur. Heart J.* 37:2315. 10.1093/eurheartj/ehw106 27222591 PMC4986030

[B19] Polonsky T. S. Ning H. Daviglus M. L. Liu K. Burke G. L. Cushman M. (2017). Association of cardiovascular health with subclinical disease and incident events: the multi–ethnic study of atherosclerosis. *J. Am. Heart Assoc.* 6:e004894. 10.1161/JAHA.116.004894 28320747 PMC5524019

[B20] Tarkin J. M. Joshi F. R. Rudd J. H. (2014). PET imaging of inflammation in atherosclerosis. *Nat. Rev. Cardiol.* 11 443–457. 10.1038/nrcardio.2014.80 24913061

[B21] Teodoro R. Scheunemann M. Wenzel B. Peters D. Deuther-Conrad W. Brust P. (2018). Synthesis and radiofluorination of novel fluoren-9-one based derivatives for the imaging of α7 nicotinic acetylcholine receptor with PET. *Bioorg. Med. Chem. Lett.* 28 1471–1475. 10.1016/j.bmcl.2018.03.081 29628323

[B22] Vieira-Alves I. Coimbra-Campos L. M. C. Sancho M. da Silva R. F. Cortes S. F. Lemos V. S. (2020). Role of the alpha7 nicotinic acetylcholine receptor in the pathophysiology of atherosclerosis. *Front. Physiol.* 11:621769. 10.3389/fphys.2020.621769 33424644 PMC7785985

[B23] Wang C. Chen H. Zhu W. Xu Y. Liu M. Zhu L. (2017). Nicotine accelerates atherosclerosis in apolipoprotein E–deficient mice by activating α7 nicotinic acetylcholine receptor on mast cells. *Arterioscler. Thromb. Vasc. Biol.* 37 53–65. 10.1161/ATVBAHA.116.307264 27834689

[B24] Wang D. Yao Y. Wang S. Zhang H. He Z.-X. (2021). The availability of the α7-nicotinic acetylcholine receptor in early identification of vulnerable atherosclerotic plaques: a study using a novel ^18^F-label radioligand PET. *Front. Bioeng. Biotechnol.* 9:640037. 10.3389/fbioe.2021.640037 33777911 PMC7994753

[B25] Wang S. Fang Y. Wang H. Gao H. Jiang G. Liu J. (2018). Design, synthesis and biological evaluation of 1,4-diazobicylco[3.2.2]nonane derivatives as α7-nicotinic acetylcholine receptor PET/CT imaging agents and agonists for Alzheimer’s disease. *Eur. J. Med. Chem.* 159 255–266. 10.1016/j.ejmech.2018.09.064 30296684

[B26] Wong D. F. Kuwabara H. Pomper M. Holt D. P. Brasic J. R. George N. (2014). Human brain imaging of α7 nAChR with [18 F] ASEM: a new PET radiotracer for neuropsychiatry and determination of drug occupancy. *Mol. Imaging Biol.* 16 730–738. 10.1007/s11307-014-0779-3 25145965 PMC5344036

[B27] Writing Group Members, Mozaffarian D. Benjamin E. J. Go A. S. Arnett D. K. Blaha M. J. (2016). Heart disease and stroke statistics-2016 update: a report from the American heart association. *Circulation* 133 e38–360. 10.1161/CIR.0000000000000350 26673558

[B28] Zhang H. Dong S. Li Z. Feng X. Xu W. Tulinao C. M. S. (2019). Biointerface engineering nanoplatforms for cancer-targeted drug delivery. *Asian J. Pharm. Sci.* 15 397–415. 10.1016/j.ajps.2019.11.004 32952666 PMC7486517

[B29] Zhao Z. He Z. Huang H. Chen J. He S. Yilihamu A. (2020). Drug-induced interstitial lung disease in breast cancer patients: a lesson we should learn from multi-disciplinary integration. *BIO Integr.* 1 82–91. 10.15212/bioi-2020-0009

