# Supplementary material for: 18F-ASEM Imaging for Evaluating Atherosclerotic Plaques Linked to α7-Nicotinic Acetylcholine Receptor
Source: Front Bioeng Biotechnol. 2021 Jul 1;9:684221. doi: 10.3389/fbioe.2021.684221 (PMC8280778; doi:10.3389/fbioe.2021.684221)
Supplement: Supplementary file 1 [file Data_Sheet_1.PDF]

Sample Name: NTBA
Lot No.: NTBA-T190901
Metod: 1H-NMR

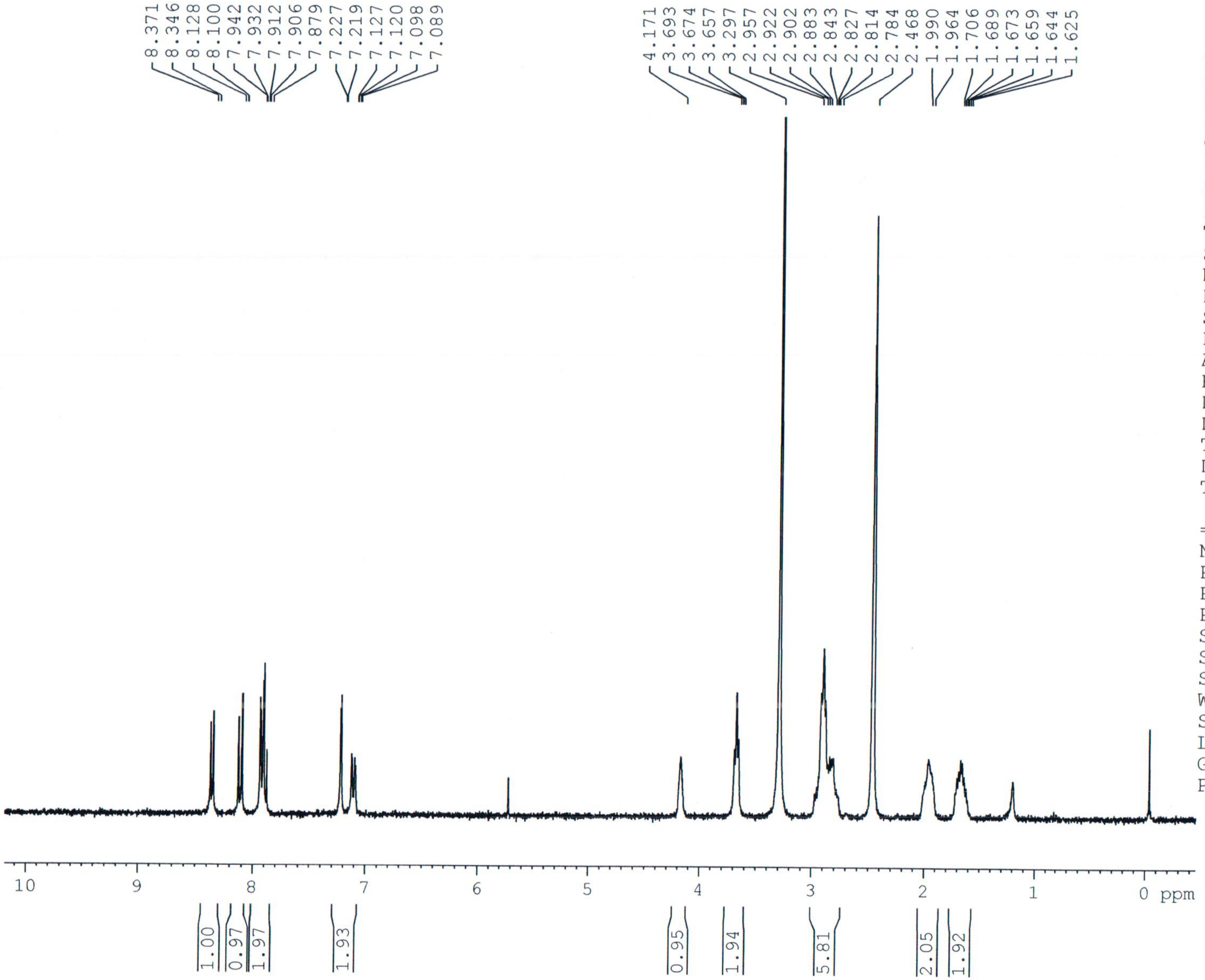

NAME	2019 team3	
EXPNO	408	
PROCNO	1	
Date_	20190929	
Time_	14.32	
INSTRUM	spect	
PROBHD	5 mm PABBO BB-	
PULPROG	zg30	
TD	32768	
SOLVENT	DMSO	
NS	48	
DS	0	
SWH	5411.255	Hz
FIDRES	0.165138	Hz
AQ	3.0278132	sec
RG	1625.5	
DW	92.400	usec
DE	6.00	usec
TE	296.2	K
D1	2.00000000	sec
TD0	1	

======== CHANNEL f1 ========

NUC1	1H	
P1	10.00	usec
PL1	-3.00	dB
PL1W	20.39832115	W
SFO1	300.1320925	MHz
SI	16384	
SF	300.1300114	MHz
WDW	no	
SSB	0	
LB	0.00	Hz
GB	0	
PC	1.00	
