# Supplementary figures and images for: 18F-ASEM Imaging for Evaluating Atherosclerotic Plaques Linked to α7-Nicotinic Acetylcholine Receptor
Source: Front Bioeng Biotechnol. 2021 Jul 1;9:684221. doi: 10.3389/fbioe.2021.684221 (PMC8280778; doi:10.3389/fbioe.2021.684221)

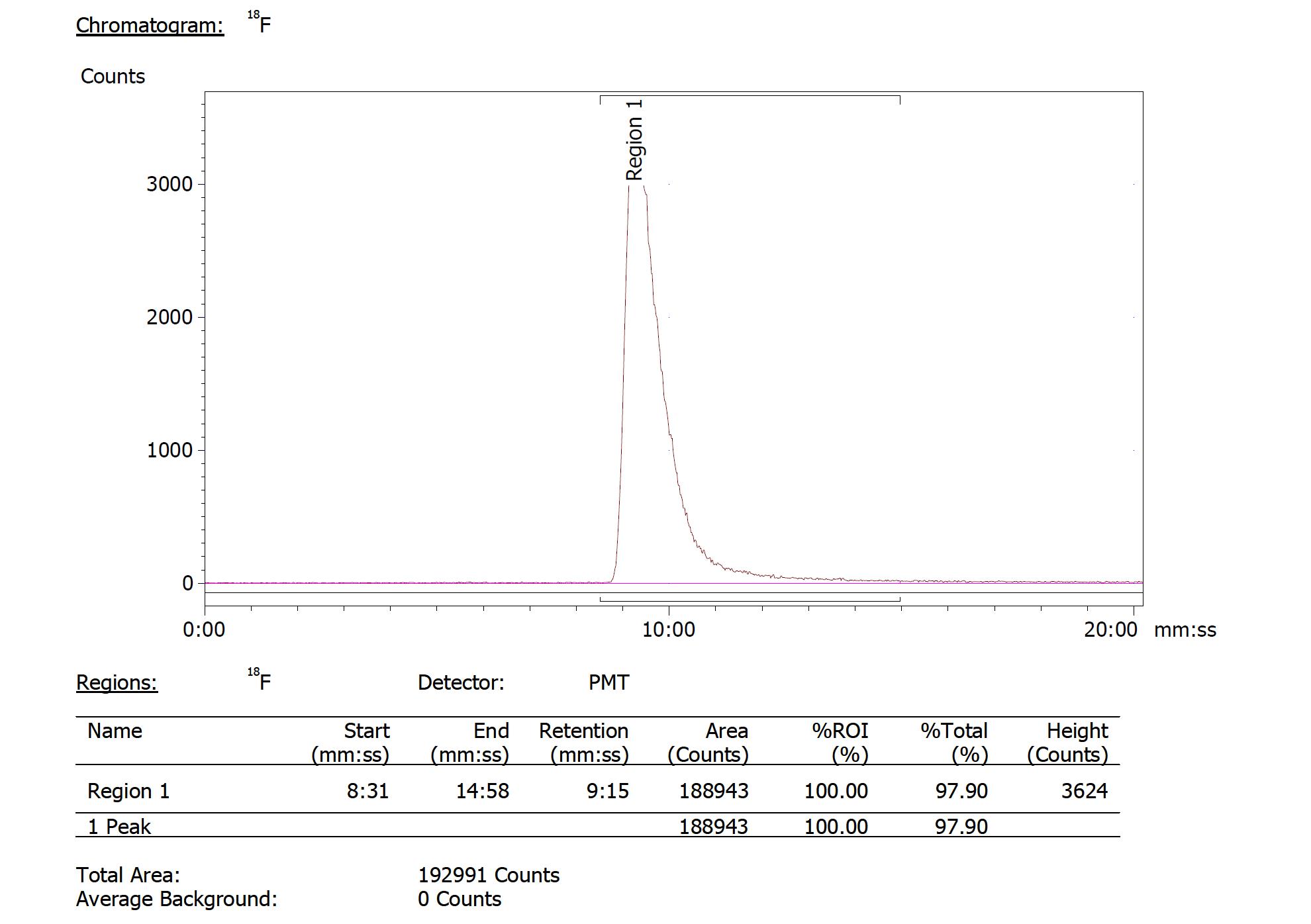

Supplement: Supplementary file 2 [file Image_1.JPEG]
