# Supplementary material for: 18F-ASEM Imaging for Evaluating Atherosclerotic Plaques Linked to α7-Nicotinic Acetylcholine Receptor
Source: Front Bioeng Biotechnol. 2021 Jul 1;9:684221. doi: 10.3389/fbioe.2021.684221 (PMC8280778; doi:10.3389/fbioe.2021.684221)
Supplement: Supplementary file 3 [file Table_1.DOCX]

The synthesis of the ASEM precursor is divided into two steps:

Step1: N-Bromosuccinimide (NBS) (1.0mmol) was added to a solution of the starting compound b (1.0mmol) in concentrated H_2_SO_4_ (3.6mL) at room temperature. After 24h, the solution was carefully poured into ice/water. The solids were filtered and washed with water and methanol. The obtained solids were recrystallized from 95％EtOH to afford bromo intermediate compounds.Yield: 77% (1.73g); ^1^H NMR (400 MHz, DMSO-d6) δ: 8.70 (d, J=8.0Hz, 1H), 8.45-8.43 (m, 2H), 8.28 (d, J=8.0Hz, 1H), 8.14-8.09 (m, 2H), HRMS calculated for C_12_H_6_BrNNaO_4_S ([M+Na]+) 361.9093; found, 361.9080.

Step2: A catalyst solution was prepared by mixing tris(dibenzylideneacetone)dipalladium (Pd(dba)_3_, 58mg, 0.063mmol) and racemic BINAP (39mg, 0.125mmol) in toluene (4mL) and heating the mixture to 90℃ for 15min. The solution was cooled and then added to a mixture of 1,4-diazabicyclo[3.2.2]nonane (200mg, 1.58mmol) and bromo intermediate compounds (0.492g, 1.58mmol), in toluene (12mL). Cs_2_CO_3_ (766mg, 2.4mmol) was then added, and the reaction mixture was flushed with nitrogen and heated overnight at 80-85℃. After cooling to room temperature, the mixture was concentrated and purified by silica gel flash chromatography (CHCl_3_/i-PrOH/Et_3_N 10:1:0.2). The ASEM precursor was obtained as a reddish solid; Yield: 55％ (80mg). ^1^H NMR (400 MHz, DMSO-d6) δ: 8.40 (d, J=4.0Hz, 1H), 8.15 (d, J=8.0Hz, 1H), 7.97 (d, J=8.0Hz, 1H), 7.94 (d, J=8.0Hz, 1H), 7.26 (d, J=4.0Hz, 1H), 7.15 (d, J=4.0Hz, 1H), 4.21 (s, 1H), 3.71 (m, 2H), 3.00-2.85 (m, 6H), 2.00 (s, 2H), 1.71 (m, 2H), HRMS calculated for C_19_H_20_N_3_O_4_S ([M+H]) 386.1169; found, 386.1150.
